# CPEB1 restrains proliferation of Glioblastoma cells through the regulation of p27^Kip1^ mRNA translation

**DOI:** 10.1038/srep25219

**Published:** 2016-05-04

**Authors:** Silvia Galardi, Massimo Petretich, Guillaume Pinna, Silvia D’Amico, Fabrizio Loreni, Alessandro Michienzi, Irina Groisman, Silvia Anna Ciafrè

**Affiliations:** 1 Department of Biomedicine and Prevention, University of Rome “Tor Vergata”, 00133 Rome, Italy; 2 Institute for Integrative Biology of the Cell, IBITECS, CEA, CNRS, Université Paris-Sud, Université Paris-Saclay, 91198, Gif-sur-Yvette cedex, France; 3 ^3^Department of Biology, University of Rome “Tor Vergata”, 00133 Rome, Italy

## Abstract

The cytoplasmic element binding protein 1 (CPEB1) regulates many important biological processes ranging from cell cycle control to learning and memory formation, by controlling mRNA translation efficiency via 3′ untranslated regions (3′UTR). In the present study, we show that CPEB1 is significantly downregulated in human Glioblastoma Multiforme (GBM) tissues and that the restoration of its expression impairs glioma cell lines growth. We demonstrate that CPEB1 promotes the expression of the cell cycle inhibitor p27^Kip1^ by specifically targeting its 3′UTR, and competes with miR-221/222 binding at an overlapping site in the 3′UTR, thus impairing miR-221/222 inhibitory activity. Upon binding to p27^Kip1^ 3′UTR, CPEB1 promotes elongation of poly-A tail and the subsequent translation of p27^Kip1^ mRNA. This leads to higher levels of p27^Kip1^ in the cell, in turn significantly inhibiting cell proliferation, and confers to CPEB1 a potential value as a tumor suppressor in Glioblastoma.

The cytoplasmic element binding protein 1 (CPEB1) is the founding member of a family of four conserved sequence-specific RNA-binding proteins (CPEB1–4) that regulate gene expression at the post-transcriptional level.^1^ CPEBs specifically bind to the cytoplasmic polyadenylation element (CPE; consensus sequence UUUUUAU) in 3′UTRs of messenger RNAs and control, along with other cellular factors, both translational repression and activation through regulation of poly(A) tail length^2^. Initially CPEBs were identified as maternal mRNA regulators, responsible of cytoplasmic polyadenylation of dormant mRNAs with short poly(A) tails and consequent induction of their translation during early embryonic development^3^,^4^. In the last years, the data about the biochemistry and biology of CPEBs has grown rather substantially and it is now clear that these proteins are key mediators of several important biological processes ranging from cell cycle control and cancer to learning and memory^5^,^6^.

Some evidences suggest a potential tumor suppressive role for CPEB1. The expression level of CPEB1 mRNA were found decreased in ovarian, melanoma and gastric cancer as well as in cell lines derived from breast, myeloma and colorectal cancer^7^,^8^,^9^,^10^,^11^. This reduction has been associated with the capacity of malignant cells to promote invasion, angiogenesis, to increase resistance to nutritional stress and to induce epithelial-to-mesenchymal transition^12^,^13^. Moreover, CPEB1-knockout human foreskin fibroblasts proliferate more rapidly, bypassing senescence and are subject to the “Warburg effect”, an aerobic glycolysis considered to be a hallmark of cancer cells^14^.

GBM is the most common and aggressive primary brain tumor. Despite interventional therapy, the overall prognosis for GBM patients remains poor^15^; thus, it is essential to understand its molecular pathogenesis, in order to provide new insight into modern therapy. Among factors whose mis-regulation has been linked to GBM progression, the cyclin-dependent kinase inhibitor p27^Kip1^ is an established prognostic marker and its expression has been inversely related to tumor grade and positively related to favorable outcome of GBM patients^16^,^17^. p27^Kip1^ plays a pivotal role in the control of cell proliferation, differentiation, and apoptosis^17^,^18^,^19^; it is considered an unusual tumor-suppressor protein, as its deregulated expression in cancer is almost exclusively due to misregulation of its gene expression at the mRNA or the protein level, rather than due to mutations in the p27^Kip1^ gene. Different mechanisms of misregulation of p27^Kip1^ expression were proposed in human gliomas. Particularly, specific microRNAs (miRNAs), a class of post-transcriptional negative regulators that control gene expression by binding to 3′UTRs, were shown to control p27^Kip1^ expression in several tumors^20^,^21^,^22^,^23^. Among miRNAs regulating p27^Kip1^, miR-221/222 were shown to negatively affect the translation of p27^Kip1^ mRNA in glioblastoma^20^,^24^ where the increased expression level of miR-221/222 clearly correlates with malignancy, as opposed to that of p27^Kip1^.

In the present study, we found that CPEB1 is significantly downregulated in human gliomas and that the restoration of its expression impairs GBM cell lines growth. We demonstrated that CPEB1 is directly involved in p27^Kip1^ expression regulation, by specifically targeting the 3′UTR of p27^Kip1^ and counteracting miR-221/222 action.

## Results and Discussion

### CPEB1 ectopic modulation affects proliferation of glioma cells

**Figure 1:**
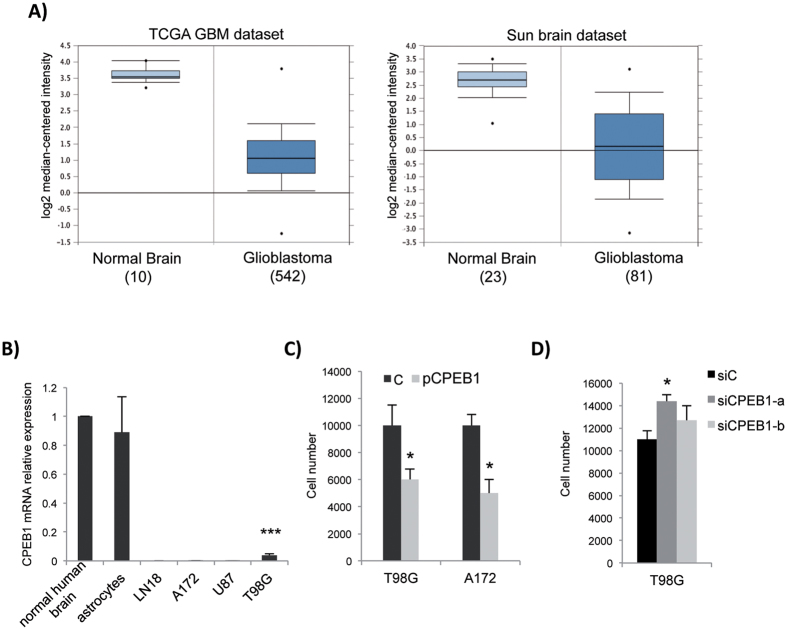
CPEB1 is downregulated in glioblastoma and affects proliferation of glioma cell lines. (**A**) Microarray data for CPEB1 gene expression from TCGA dataset consisting of normal (n = 10) and GBM (n = 542) samples; or from “Sun brain” dataset consisting of normal (n = 23) and GBM (n = 81) samples, are shown as a box-plots. A non-parametric t test was performed to calculate p values as indicated in the text. The horizontal line in each plot represents the mean value. (**B**) Total RNA was extracted from A172, LN18, U87 and T98G glioblastoma cell lines, or from cultured human astrocytes and endogenous CPEB1 mRNA expression was analysed by RT-qPCR. CPEB1 mRNA expression in all samples is reported as compared to that in “normal human brain” (Ambion-Thermofisher #AM7962), set as = 1. ANOVA test in combination with Dunnett’s test were performed to calculate p values. (**C**) Cells were transfected with the empty plasmid (C) or pCPEB1 plasmid^7^ and, after 48 h, viable cells were counted using a hemocytometer. Student’s t-test was performed. D, T98G cells transfected with siRNAs against CPEB1^7^ or a non-targeting control siRNA (siC) were analysed as in (**C**). In this and all the subsequent figures, *p < 0.05, **p < 0.01, ***p < 0.001. All experiments were performed three times (biologic replicates). All histograms in all figures refer to mean ± S.D.

To evaluate the possible role of CPEB1 in glioma malignancy, we searched the Oncomine database^25^ for CPEB1 expression in large glioblastoma datasets. Among them, in the Cancer Genome Atlas (TCGA) showing the distribution of CPEB1 mRNA expression in 542 GBM tissues versus 10 normal brain tissues, we found that the CPEB1 mRNA levels in the GBM group were significantly lower than those in the normal brain group (Log_2_FC = −5.607, p = 5.68E-15, Fig. 1A). This observation was confirmed in another independent dataset, the “Sun brain”, comparing 81 GBM samples to 23 healthy controls (Log_2_FC_GBMvsCTRL_ = −5.360, p = 5.10E-21, Fig. 1A). We then applied real-time PCR to quantitatively analyse endogenous CPEB1 expression in a panel of human GBM cell lines and we determined that CPEB1 was not expressed in LN-18, A172, and U87MG cell lines and it was 20-fold less expressed in T98G cell line as compared with normal human brain and astrocytes (Fig. 1B).

To elucidate the function of CPEB1 in GBM, we ectopically altered its expression and found that the proliferation rate of glioma cells was strongly modified. Fig. 1C shows the results of a proliferation assay where the viability of T98G and A172 cells transfected with the empty vector, was compared with that of cells transfected with a plasmid expressing FLAG-tagged CPEB1 (pCPEB1, Fig. S1A): the expression of CPEB1 induced on average a 2-fold decrease in the proliferation rate of both cell lines. Consistently, when T98G cells, which express detectable levels of endogenous CPEB1, were siRNA knocked down for CPEB1 (Fig. S1B) and compared with the control, their proliferation rate increased (Fig. 1D).

Altogether, our observations are in agreement with a meta-analysis that points out how CPEB1 mRNA is most highly expressed in the brain, and its expression is reduced in cancers in this tissue^26^,^27^, and with a recent study where it was found that the expression profile of CPEB1 negatively correlated with overall survival of glioma patients^28^. Interestingly, this study observed that CPEB1 overexpression decreased proliferation and infiltration of glioma stem cells in an orthotopically implanted animal model^28^.

### CPEB1 regulates p27^Kip1^ expression by binding a specific CPE in the 3′UTR of its mRNA

**Figure 2:**
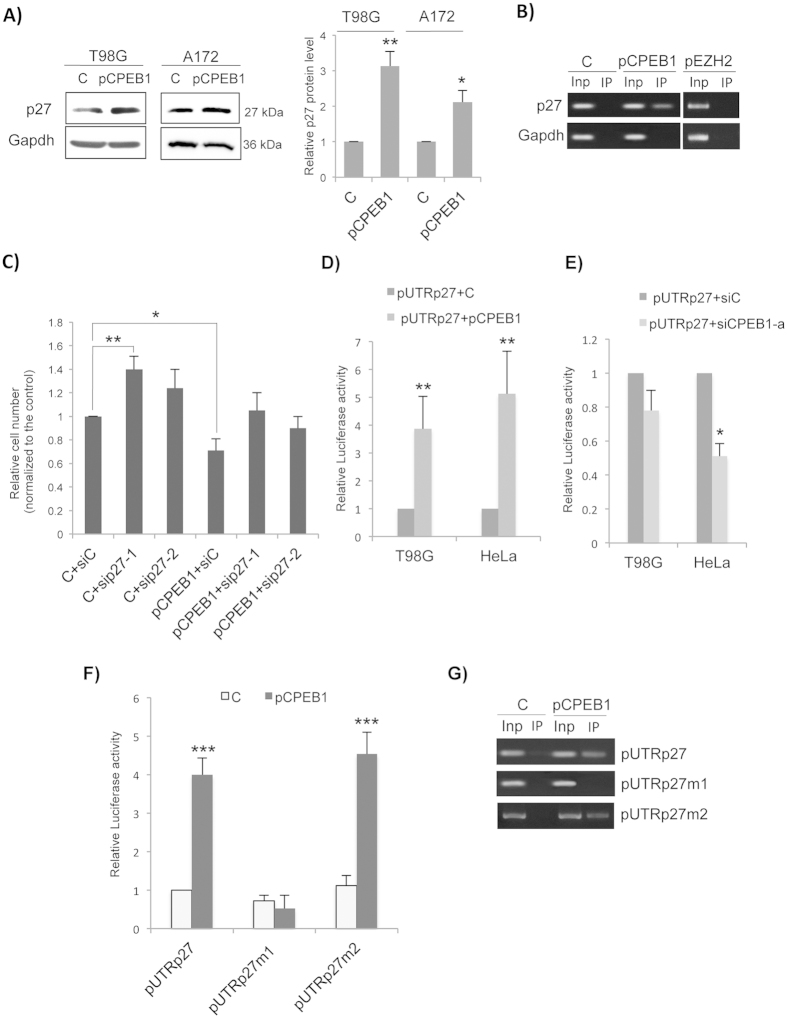
CPEB1 directly interacts with the 3′UTR of p27^kip1^. (**A**) T98G and A172 cells were transfected with the empty vector (C) or pCPEB1. After 48 h, the whole-cell lysates were run on the same gel, blotted onto nitrocellulose and the membrane cut to allow probing with antibodies against p27 or Gapdh. Relative signal intensities (right panel) for p27 in the Western blots normalized to the Gapdh loading control, are included for comparison of p27 levels. Bars represent average +/− SD of three independent experiments. Student’s t-test was utilized to calculate p values. (**B**) Cell lysates from T98G cells trasfected with the empty vector (**C**), pCPEB1 or pEZH2, were subjected to RIP assay with Flag antibody. P27 and GAPDH mRNA levels were detected using RT-PCR as shown in the representative cropped gel. (**C**) Proliferation assay of HeLa cells co-transfected with pCPEB1, siRNAs against p27 and relative controls was performed as in Fig. 1C. ANOVA test in combination with Dunnett’s test were performed to calculate p values, comparing the mean of each column with the mean of the control column. (**D**) pUTRp27 luciferase and a *renilla* control luciferase constructs were co-transfected with the empty vector (**C**) or pCPEB1. After 48 h from transfection, T98G and HeLa cells were harvested and assayed with Dual Luciferase Assay (Promega) according to the manufacturer’s instructions. Relative luciferase activity is the ratio between *firefly* luciferase and *renilla* control luciferase. Student’s t-test was utilized to calculate p values. (**E**) pUTRp27 luciferase constructs were co-transfected with siRNA negative control (siC) or siRNA against CPEB1, and cells were treated as in (**D**). (**F**) HeLa cells were transfected with the indicated luciferase constructs in the presence of empty vector (C) or pCPEB1 and processed as in (**D**). (**G**) Protein complexes extracted from HeLa cells, transfected with the indicated luciferase constructs, were immunoprecipitated and treated as indicated in (**B**). The cDNA was amplified by specific oligos corresponding to the *Firefly* luciferase mRNA.

To better understand the anti-proliferative role of CPEB1, we performed a bio-informatics search for target transcripts involved in GBM progression that could potentially be modulated by CPEB1. Searching for the most promising predicted targets of CPEB1 that can influence cell proliferation we discovered that the 3′UTR of p27^Kip1^ mRNA harbours 2 putative CPE sequences, located at nucleotides 1336–1341 and 1849–1854 (Fig. S1C). To check if CPEB1 could affect p27^Kip1^ expression in glioma cells, we analysed the consequences of the transfection of a FLAG-CPEB1 expression vector (pCPEB1) on p27^Kip1^ protein expression level; Fig. 2A shows that the ectopic expression of CPEB1 increases the endogenous levels of p27^Kip1^ in both T98G and A172 cell lines. Since CPEB1 acts as a sequence-specific RNA-binding protein, we analysed the possible interaction between CPEB1 and p27^Kip1^ mRNA by using RNA-binding Protein-immunoprecipitation (RIP) experiments. We transfected T98G cells with pCPEB1 and we performed FLAG-IP followed by RT-PCR with primers specific for p27^Kip1^ mRNA. We found that the mRNA was co-precipitated with FLAG-CPEB1 and that the RIP was specific since the non-CPE-containing mRNA encoding GAPDH was not co-precipitated (Fig. 2B). Thus, we determined that p27 ^Kip1^ mRNA is in the CPEB1 complex. The specificity of the interaction was confirmed when we performed RIP experiments with the unrelated FLAG tagged EZH2, a protein involved in epigenetic modulation of chromatin (Fig. 2B, right panel).

To check if p27^Kip1^ is an essential target through which CPEB1 modulates cell proliferation, we decided to perform siRNA knock down of p27^Kip1^ in the presence or absence of pCPEB1, and to verify if we can obtain a rescue effect on the proliferation rate of CPEB1-over-expressing cells. We transfected pCPEB1 into T98G cells previously treated with anti-p27 siRNAs (sip27–1 Fig. S1D), and we observed that, 48 h post-transfection, the proliferation rate was comparable to that of control cells, indicating that p27^Kip1^ siRNA was able to rescue the effect of CPEB1 overexpression on cell proliferation (Fig. 2C). Altogether, our results suggest that CPEB1 can influence cell proliferation, at least in part by modulating p27^Kip1^ expression.

In an attempt to determine the molecular mechanisms of the interaction between CPEB1 and the 3′UTR of p27^Kip1^ mRNA, we utilized a reporter construct containing the wild-type 3′UTR of p27^Kip1^ cloned downstream of the firefly luciferase cDNA (pUTRp27)^21^. When we co-transfected pCPEB1 and pUTRp27, we observed that the overexpression of CPEB1 strongly increased the luciferase expression, measured as relative luciferase activity, in both T98G glioblastoma cells and in the unrelated HeLa cells, which endogenously express CPEB1 (Fig. 2D). On the contrary, when we knocked down CPEB1, the relative luciferase expression was reduced (Fig. 2E). These results support CPEB1 regulation of p27^Kip1^ mRNA through its 3′UTR, which probably occurs via the CPE elements in p27^Kip1^ mRNA 3′UTR. To verify if the two putative CPE identified were functional, we mutated the pUTRp27 reporter construct at either the first or at the second CPE site (pUTRp27m1 and pUTRp27m2, respectively). We found that pUTRp27m1 is not sensitive to the action of CPEB1, while pUTRp27m2 behaved as the wt construct; in fact its luciferase activity significantly responded to the presence of CPEB1 (Fig. 2F). We then further investigated the role of the first CPE site with respect to CPEB1 binding, by performing RIP experiments where pCPEB1 was co-transfected with pUTRp27, pUTRp27m1 or pUTRp27m2 constructs. Figure 2G shows that CPEB1 was able to bind the mRNAs derived from pUTRp27 and pUTRp27m2 but not with the mRNA derived from pUTRp27m1, indicating that only the first CPE site was responsible of the CPEB1-p27^Kip1^ mRNA interaction.

### CPEB1 counteracts miR-221/222 repression of p27^Kip1^

**Figure 3:**
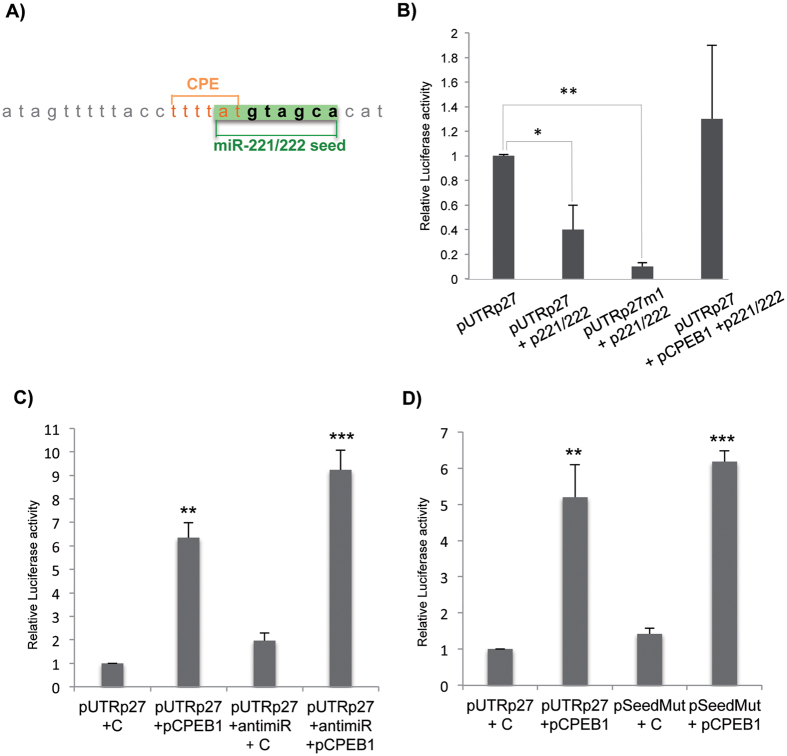
CPEB1 competes with miR221/222 binding at an overlapping site in the 3′UTR of p27^kip1^. (**A**) p27 mRNA 3′UTR putative sites targeted by CPEB (orange) and by miR-221/222 seed sequence (green). (**B**) pUTRp27 or pUTRp27m1 were co-transfected with a plasmid expressing miR221/222 (p222/221) and pCPEB1 (last bar). After 48 h, HeLa cells were subjected to luciferase assay as in Fig. 2D. (**C**) pUTRp27 was co-transfected with pCPEB1 and/or LNA antisense oligonucleotides directed against endogenous miR-221 and miR-222 (antimiR). After 48 h, cells were treated as in (**B**). (**D**) HeLa cells were transfected with either pUTRp27 or pSeedMut together with pCPEB1 or an empty vector. After 48 h, cells were subjected to luciferase assay as in Fig. 2D. ANOVA test in combination with Dunnett’s test were performed to calculate p values. The mean of each column was compared with the mean of the control column.

Notably, the first CPE in p27^Kip1^ mRNA 3′UTR partially overlaps with the seed sequence recognized by miR-221 and miR-222 (Fig. 3A). MiR-221 and miR-222 (miR-221/222) are two polycistronic miRNAs sharing the same seed sequence, widely overexpressed in a large variety of human cancers, where they were shown having oncogenic roles via the downregulation of several tumor suppressors^20^,^21^,^22^,^23^. In our previous work, we demonstrated that miR-221/222 are overexpressed in glioblastoma, where they downregulate p27^Kip1^ by impairing the translation of its mRNA^20^,^24^. In the last few years an ever-growing number of functional interactions between miRNAs and RBPs have been discovered, pointing out a new level of complexity of gene expression regulation^29^. For instance, in a recent paper, we demonstrated the interaction between CPEB1 and miR-580 in an *in vitro* model of breast cancer progression where we showed that the protein and the miRNA act as negative regulators of TWIST1 expression in a sequence-specific and additive/cooperative manner^7^.

To investigate the possibility that CPEB1 could interplay with miR-221/222 in the regulation of p27^Kip1^ expression, we co-transfected pUTRp27 in HeLa cells with a plasmid expressing miR-221/222 (pmiR221/222)^20^, with (or without) pCPEB1. When we co-expressed miR-221/222 and pUTRp27, we observed, as expected, that the ectopic expression of miR-221/222 significantly reduced luciferase activity of the reporter gene; however, the addition of pCPEB1 impaired mir-221/222 action and restored the pUTRp27 luciferase activity to that of control levels (Fig. 3B). Interestingly, when we measured the miR-221/222 effect on the luciferase activity of pUTRp27m1 (the only mutant unable to bind CPEB1), we found that the resulting repression was significantly enforced (Fig. 3B), possibly due to the abolishment of the interaction of endogenous CPEB1 with p27^Kip1^ 3′UTR. These results indicate that CPEB1 is able to counteract miR-221/222 action, probably through the binding to the CPE sequence. Remarkably, in a different context, miR-221/222 regulation of p27^Kip1^ expression was shown to be interfered by Dnd1, a RBP required for germ cell survival and migration in zebrafish^30^. Kedde and collaborators showed that Dnd1 counteracts the function of miR-221/222, by binding the p27^Kip1^ 3′UTR and hampering miRNAs from associating with their target site^31^. Dnd1 effects are miRNA-dependent since the protein is not able to modulate p27^Kip1^ expression in a miR-221/222 depleted context. To test if also CPEB1 activity depends on miR-221/222 function, we knocked down the two miRNAs in HeLa cells that express them endogenously, by the use of LNA antisense oligonucleotides that we employed in a previous work, to target both miR-221 and miR-222^20^. Figure 3C confirms that the introduction of pCPEB1 or inhibition of miR-221/222 elevated the luciferase activity of pUTRp27; the co-introduction of both pCPEB1 and LNA oligoes caused a further increase in pUTRp27 luciferase activity, demonstrating that CPEB1 is able to act also in the absence of miR-221/222. These results were confirmed when we measured the luciferase activity of pSeedMut, a construct derived from pUTRp27 where we mutated the nucleotides of the seed sequence that do not overlap with the nucleotides of CPE site (in bold, Fig. 3A). The results depicted in Fig. 3D show that the abolishment of miR-221/222 binding to the region containing the CPE, tends to strengthen the stimulating action of CPEB1, even if in a non-statistically significant way, that is anyway evident both in the presence (pUTRp27+pCPEB1) or in the absence (pSeedMut+pCPEB1) of miR-221/222 binding.

### CPEB1 expression stimulates translation of p27^kip1^ transcript

**Figure 4:**
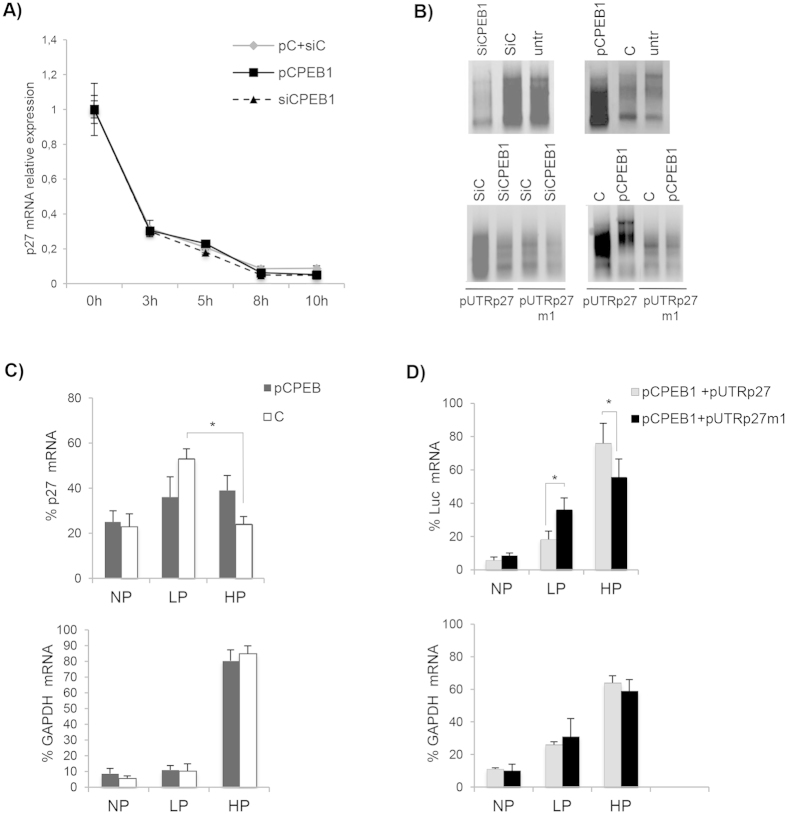
CPEB stimulates p27 expression by promoting the elongation of polyA tail and translation efficiency. (**A**) HeLa cells transfected with either empty vector, or pCPEB1 or siRNAs against CPEB1 were treated with Actinomycin D (10 μg/ml). RNA was isolated at various time points after the treatment and analyzed by RT-qPCR. (**B**) HeLa cells were transfected as indicated in the panels and after 48 h, total RNA was isolated and subjected to PAT assays. (**C**) Cells transfected with control vector or pCPEB1 were lysed and cytoplasmic extracts were subjected to polysome gradient. RNA extracted from gradients collected in fraction HP (Heavy Polysomes), fraction LP (Light Polysomes) and fraction NP (Non-Polysomes) was reverse transcribed into cDNA and analysed by qPCR with primers specific for p27 (upper panel) or GAPDH (lower panel). The final result was reported as the percentage of RNA in each fraction. (**D**) Cells co-transfected with pCPEB1 and either pUTRp27 or pUTRp27m1 were treated as in (**C**). The RT-qPCR was performed with primers specific for the *Firefly* luciferase mRNA. ANOVA test in combination with Tukey’s multiple comparisons test were performed to calculate p values in (**C**,**D**).

To further clarify the molecular mechanisms of CPEB1 in p27^Kip1^ regulation, we examined if p27^Kip1^ mRNA stability was affected as a consequence of CPEB1 ectopic modulation. Cells transfected with pCPEB1, anti-CPEB1 siRNA or control were treated with Actinomycin D and total RNA was isolated at the indicated time points and analysed by RT-qPCR. As shown in Fig. 4A, the ectopic modulation of CPEB1 did not change the half-life of p27^Kip1^ mRNA, indicating that CPEB1 action is not exerted through the modulation of p27^Kip1^ mRNA stability. We then assumed that CPEB1 might affect p27^Kip1^ expression by modulating the translation of its mRNA. Since CPEB1 was originally identified as a polyadenylation factor^32^ and since polyadenylation strongly stimulates translation, we asked whether the polyadenylation status of p27^Kip1^ mRNA was altered by CPEB1 modulation. We performed a PCR poly(A) tail (*PAT*) assay on both endogenous p27^Kip1^ mRNA and luciferase mRNAs, derived from pUTRp27 and pUTRp27m1 constructs in cells transfected with pCPEB1, anti-CPEB1 siRNAs, or controls. Ectopic expression of CPEB1 stimulated polyadenylation of both endogenous p27^Kip1^ mRNA and exogenous luciferase construct attached to the wild type p27^Kip1^ 3′UTR, but it did not affect the polyadenylation of the mRNA transcribed from pUTRp27m1. Coincidently, siRNA-mediated knock down of CPEB1 decreased the polyadenylation of both endogenous p27 mRNA and pUTRp27 wt but not that of the pUTRp27m1 derived mRNAs (Fig. 4B). Since the first CPE is located quite far from the 3′ end of p27 3′UTR and polyadenylation signal (AAUAAA), and it is not very clear how, from that distal site, it could influence the polyadenylation process, we decided to verify if any potential secondary structure might shorten the distance between this CPE and the polyadenylation signal. Indeed, a secondary structure prediction of p27^Kip1^ mRNA 3′UTR obtained by an RNA structure prediction algorithm (http://mfold.rna.albany.edu/), computed a few conformations where the distance between the first CPE and the AAUAAA sequences is around 40 nucleotides (Fig. S2A), a distance potentially allowing the modulation of the polyadenylation process by the CPE sequence.

As poly(A) lengthening is expected to result in a more efficient translation of p27^Kip1^ mRNA, we examined the polysomal distribution of p27^Kip1^ mRNA in extracts of control or CPEB1-expressing cells (Fig. S2B). We found that while in control conditions p27 mRNA was enriched in the light polysomes (LP, associated with low rates of translation) fraction compared to the heavy polysomes (HP, associated with high translation rates), in the presence of exogenously overexpressed CPEB1 this difference was abolished, and p27^Kip1^ mRNA appeared equally distributed in the two fractions (Fig. 4C). We think that this result can be explained by an increase in p27^Kip1^ mRNA translation efficiency in the presence of CPEB1. To corroborate this hypothesis, and specifically check the role of CPEB1 binding site in p27 mRNA 3′UTR, we compared polysomal distribution of the mRNA expressed by pUTRp27, and pUTRp27m1. In the LP fraction, the mRNA harbouring the mutated form was more represented compared to the wild type one (Fig. 4D) while in the HP fraction, the opposite was true, the wild type 3′UTR enriched compared to the mutated one. This difference is statistically significant and indicates that CPEB1 enhances p27^Kip1^ mRNA translation by binding the first of the CPE sites present in p27^Kip1^ mRNA 3′UTR.

Altogether, our results expand the comprehension of the regulatory network that controls p27^Kip1^ expression, by highlighting a novel crosstalk between the structural components of p27^Kip1^ mRNA and the specific trans-acting factors CPEB1 and miR221/222.

Moreover, we provide new insights into the role of CPEB1 in the proliferation of gliomas that encourage further studies to explore the potential of CPEB1 as a therapeutic tool in GBM.

## Methods

### Cell lines, treatments and transfection

LN18, A172, U87MG and T98G cell lines (ATCC, Rockville, MD, USA) were cultured in Dulbecco’s modified Eagle’s medium supplemented with 10% fetal bovine serum (Invitrogen). Human astrocytes (Clini Sciences, Italy) were cultured in astrocyte medium (Clini Sciences, Italy) in a humidified atmosphere containing 5% CO_2_ at 37 °C. Transfections were performed by Lipofectamine 2000 reagent (Invitrogen) using plasmid DNA or siRNAs in Opti-MEM I (Invitrogen), as recommended by the manufacturer. For DNA transfections, reporter plasmids were co-transfected with a 1:10 relative amount of the plasmid pEGFP-C3 (Clontech), to monitor transfection efficiency. For siRNA transfections, siRNAs were co-transfected with a 1:10 relative amount of fluorescent siRNA control. Cells were analyzed by fluorescence microscopy 48 h after transfection to calculate the transfection efficiency.

### RNA isolation and Quantitative Real Time Polymerase Chain Reaction (RT-qPCR)

Total cellular RNA was harvested with TRIzol LS Reagent® (Life Technologies), treated with DNase (Promega, Madison, WI, USA) and reverse transcribed with MultiScribe reverse transcriptase (Life Technologies), according to the manufacturers’ protocols. RT-qPCR was performed on the Applied Biosystems StepOne plus PCR System with SYBR Select Master Mix (Life Technologies) and the following primers: CPEB1, 5′-TTTCAAGCCTTCGCATTTCCC-3′ and 5′-GGACCCAACGCCCATCTTTA-3′; GAPDH, 5′-GTGTTCCTACCCCCAATGTGT-3′ and 5′-ATTGTCATACCAGGAAATGAGCTT-3′; p27, 5′-CTAACTCTGAGGACACGCATTT-3′ and 5′-TGCAGGTCGCTTCCTTATTC-3′.

### Western blot analysis

Total protein extract were separated on 10% SDS-PAGE gels and analyzed by standard immunoblotting analysis with appropriate primary (anti-FLAG, F7425, Sigma-Aldrich; anti-p27 antibody, AB3003,Chemicon; anti-GAPDH, MAB374, Millipore) and secondary antibodies and Pierce ECL substrate (Thermo Scientific). Bands were quantified with ImageJ 1.34 or OptiQuant 3.1 Packard Instrument software.

### Luciferase reporter constructs and luciferase assays

The pUTRmut-p27 constructs were generated by inverse PCR of pUTR-p27 and the CPE sites were replaced with EcoRI restriction enzyme recognition sites. Primers 5′-GAATTCGTAGCACATAAACTT-3′ and 5′-GAATTCGGTAAAAACTATATACAC-3′ were used to obtain the clone pUTRp27m1, mutated in the first CPE, and primers 5′-GAATTCGTTGACAAAATTTTCTC-3′ and 5′-GAATTCGCAGATGGTCAATTCTTA-3′ were used to obtain the clone pUTRp27m2 mutated in the second CPE. The PCR products were digested by EcoRI (New England Biolabs) and ligated by T4 DNA ligase (Fisher scientific). pSeedMut was generated as pUTRmut-p27 constructs; the nucleotides of the seed sequence that do not overlap with the nucleotides of CPE were replaced with EcoRI restriction enzyme recognition sites (primers: 5′-GAATTCCATAAACTTTGGGGA-3′ and 5′-GAATTCATAAAAGGTAAAAAACTA-3′). All constructs were confirmed by sequencing. The Dual-Luciferase reporter assay system (Promega) was used; cells were transfected with each reporter construct and the TK-*Renilla* luciferase plasmid that was used as a transfection control. Luciferase detection was performed 48 h after reporter construct transfection. Expression was calculated as the relative Firefly luciferase activity normalized with respect to the activity of transfection control *Renilla* luciferase.

### Proliferation assay

Cells were trypsinized with 0.25% trypsin (HyClone) at 37 °C for 5 min. The cell suspension was thoroughly homogenized with a micropipette and aliquots of 10 ml were used for counting on a hemocytometer (Bright-Line; Hausser Scientific, Horsham, PA) in combination with the trypan blue dye. Team of two individuals counted triplicate samples from three identical sample sets.

### RIP assay

Cell lysate was incubated with antibody/beads for 18 h at 4 °C. Protein-RNA complexes bound to beads were washed three times in NT2 buffer [50 mM Tris-HCl (pH 7.4), 300 mM NaCl, 1 mM MgCl2, 0.05% Nonidet P-40 (NP40), 1 × PIC, RNase inhibitor], and then treated with DNase I for 15 min at 37 °C and further washed twice with NT2 buffer. Co-precipitated RNA was recovered by phenol-chloroform extraction and ethanol precipitation and subjected to RT-PCR analysis.

### PAT assay

Total cellular RNA was reversed transcribed with MultiScribe reverse transcriptase (Life Technologies), using oligo(dT) anchor primer (5′-GCTCCGCGGCCGCGTTTTTTTTTTTTTTT-3′), and subsequent PCR was conducted with anchor primer (5′AAAAACGCGGGCCGCGGAGC-3′) and specific primer for p27 (5′-GCCAATTATTGTTACACATT-3′) located near the 3′ end of the p27 3′UTR.

### Polysome gradient and analysis

Cells were lysed in TNM buffer (Tris-HCl pH 7.5 10 mM, NaCl 10 mM MgCl_2_ 10 mM, DTT 10 mM, Triton-DOC 1%, RNAsIN 0,13 U/μl). After incubation for 5 min in ice, the extracts were centrifuged at a 13000 rpm in a bench microcentrifuge for 5 min at 4 °C. The supernatant (cytoplasmic extract) was loaded onto a 15–50% linear sucrose gradient containing 30 mM Tris-HCl (pH 7.5), 100 mM NaCl and 10 mM MgCl_2_.

Gradients were centrifuged in a Beckman SW 41 rotor for 110 min at 37000 g. After centrifugation, gradients were collected in 3 fractions while monitoring the absorbance at 260 nm: fraction HP (Heavy Polysomes), fraction LP (Light Polysomes) and fraction NP (Non Polysomes). SDS and glycogen were added to the fractions to a final concentration of 1% and 0,01 μg/μl, respectively and RNA was extracted with the proteinase K method. In addition, 10 pg of an *in vitro* synthesized RNA (M7) was added to each fraction to be used as reference. The same aliquot of RNA from each fraction (1/3–1/5) was reverse transcribed into cDNA and part of the reaction was used in qPCR with primers specific for p27 or luciferase sequences. Amount of RNA in each fraction was calculated with the 2^−∆CT^ method using M7 RNA as reference. The final result was reported as percentage of RNA in each fraction. M7 RNA primers: forward 5′-GGCGAATTGGGCCCGACGTC-3′ reverse 5′-TGGGCTTCACGATCTTGGCG-3′

### Statistical analysis

Values are expressed as mean § SEM. Each group consisted of at least 3 independent trials for each condition studied. The statistical significance of the differences was determined using the Student’s t-test (for 2 groups) or one-way ANOVA (for more than 2 groups) followed by Student-Newman-Keuls post-hoc.analysis, by using Graphpad 6.0 software. Significance was set at p < 0.05.

## Additional Information

**How to cite this article**: Galardi, S. *et al.* CPEB1 restrains proliferation of Glioblastoma cells through the regulation of p27^Kip1^ mRNA translation. *Sci. Rep.* **6**, 25219; doi: 10.1038/srep25219 (2016).

## Supplementary Material

Supplementary Information
