# Supplementary material for: CPEB1 restrains proliferation of Glioblastoma cells through the regulation of p27Kip1 mRNA translation
Source: Sci Rep. 2016 May 4;6:25219. doi: 10.1038/srep25219 (PMC4855225; doi:10.1038/srep25219)
Supplement: Supplementary Information [file srep25219-s1.pdf]

CPEB1 restrains proliferation of Glioblastoma cells through the regulation of p27^{Kip1} mRNA translation

Silvia Galardi[1*], Massimo Petretich[2,3], Guillaume Pinna[2], Silvia D'Amico[3], Fabrizio Loreni[3], Alessandro Michienzi[1], Irina Groisman[2,5] and Silvia Anna Ciafrè[1,5].

[1] Dept. of Biomedicine and Prevention, University of Rome "Tor Vergata", 00133 Rome, Italy

[2] Institute for Integrative Biology of the Cell, IBITECS, CEA, CNRS, Université Paris-Sud, Université Paris-Saclay, 91198, Gif-sur-Yvette cedex, France

[3] Present address. Developmental Biology Unit, European Molecular Biology Laboratory, Heidelberg, Germany

[4] Dept. of Biology, University of Rome "Tor Vergata", 00133 Rome, Italy

[5] These authors contributed equally to this work

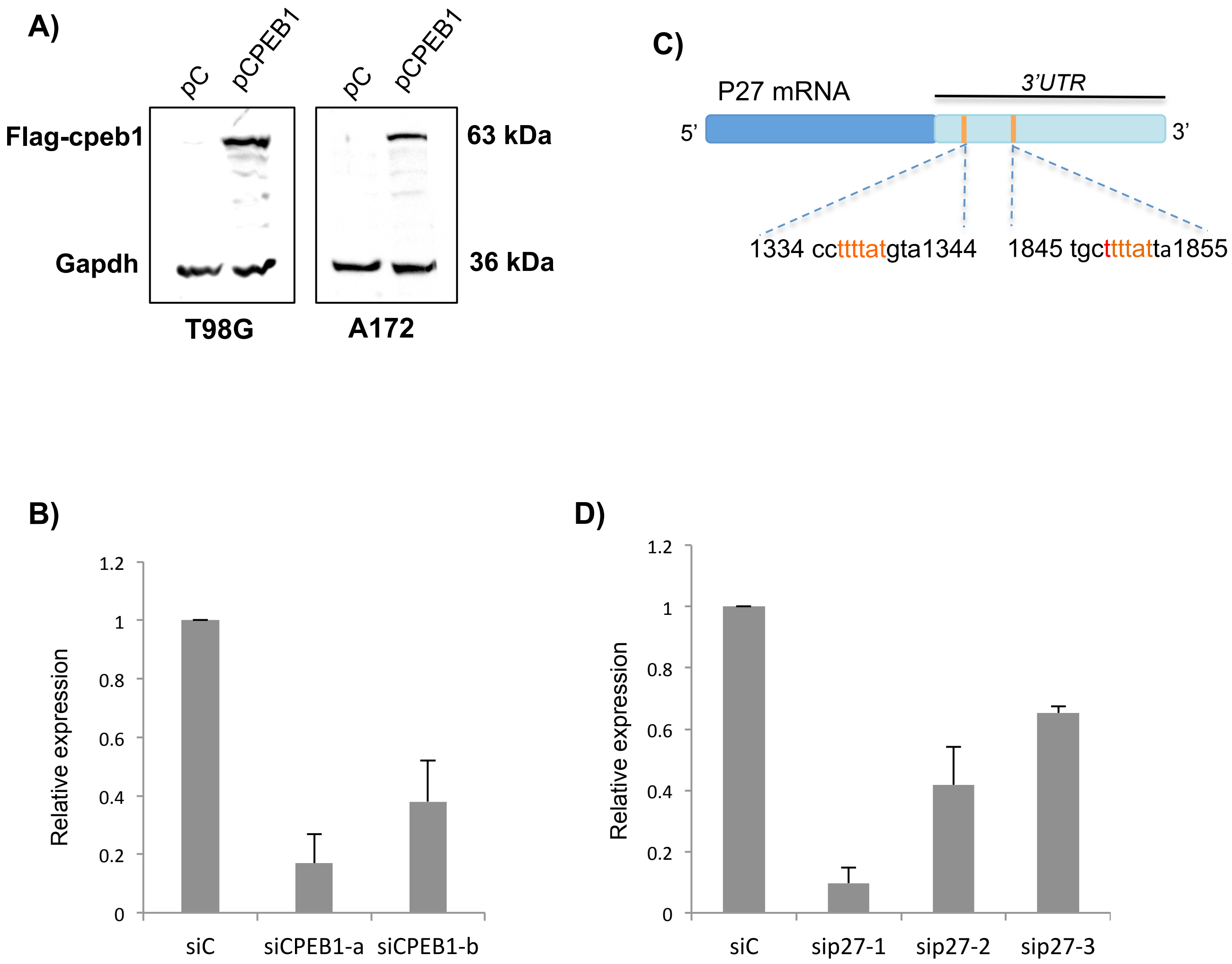

Supplementary Figure 1. A, T98G and A172 cell lines were transfected with pC or pCPEB1 and after 48h, total cell lysates were separated by 10% SDS-PAGE followed by immunoblotting with anti-CPEB1 and anti-GAPDH antibodies to measure CPEB1 expression level. B, T98G cells transfected with siC or two different siRNAs against CPEB1 were analysed by qRT-PCR to measure CPEB1 mRNA relative expression level. C, p27 mRNA 3'UTR putative sites targeted by CPEB1 (orange). D, Cells transfected with siC or three different siRNAs against p27 were analysed by qRT-PCR to measure p27 mRNA relative expression level.

A)

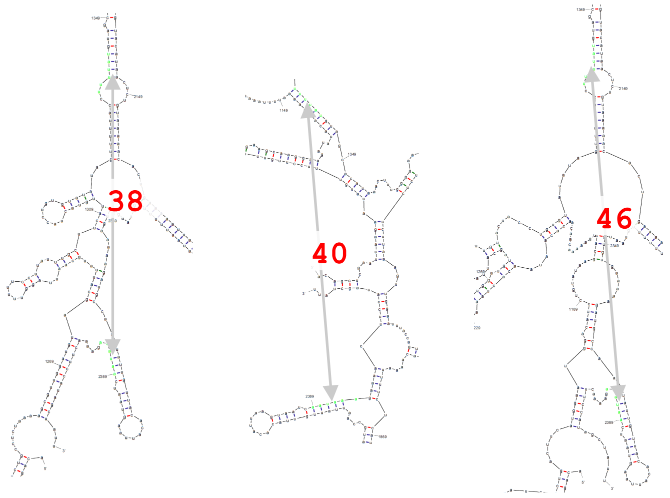

B)

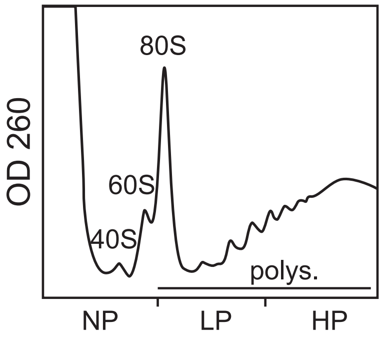

Supplementary Fugure 2. A, secondary structure prediction of p27 mRNA 3'UTR . B, a representative polysomal profile obtained while monitoring the optical density at 260 nm
